# Longitudinal EEG model detects antisense oligonucleotide treatment effect and increased UBE3A in Angelman syndrome

**DOI:** 10.1093/braincomms/fcac106

**Published:** 2022-04-26

**Authors:** Elizabeth R. Spencer, Wen Shi, Robert W. Komorowski, James P. Gilbert, Lauren M. Ostrowski, Lynne M. Bird, Ronald Thibert, Channa Bao, Fiona Molloy, Michael Calhoun, Samir Koirala, Paymaan Jafar-nejad, Frank Rigo, Mark A. Kramer, Catherine J. Chu

**Affiliations:** 1 Department of Mathematics and Statistics, Boston University, 02215 Boston, MA, USA; 2 Department of Neurology, Massachusetts General Hospital, 02114 Boston, MA, USA; 3 Harvard Medical School, 02115 Boston, MA, USA; 4 Biogen Inc, 02142 Cambridge, MA, USA; 5 School of Medicine, University of California, 92092 San Diego, CA, USA; 6 Department of Pediatrics, University of California, 92093 San Diego, CA, USA; 7 Ionis Pharmaceuticals, 92010 Carlsbad, CA, USA

**Keywords:** Angelman syndrome, antisense oligonucleotide, biomarkers, EEG, UBE3A

## Abstract

Angelman syndrome is a neurodevelopmental disorder caused by deficiency of the maternally inherited *UBE3A* gene in neurons. Antisense oligonucleotide therapies are under development to reinstate UBE3A protein production. Non-invasive biomarkers to detect target engagement and treatment response are needed to support clinical trials. Delta power measured in the scalp EEG is a reliable biomarker for Angelman syndrome but varies widely across individuals and throughout development, making detection of a treatment effect using single measurements challenging.

We utilized a longitudinal dataset of 204 EEG recordings from 56 subjects with Angelman syndrome to develop a natural history model of delta (2–4 Hz) power, with predictors of age, elapsed time, and relative delta power at an initial recording. Using this model, we computed the sample and effect sizes needed to detect a treatment effect in a human clinical trial with 80% power. We applied the same model structure to a mouse model of Angelman syndrome (*n* = 41) to detect antisense oligonucleotide-mediated treatment effects on absolute delta activity and *Ube3a* expression. In humans, delta power at a second time point can be reliably predicted using the natural history model. In mice, a treatment effect can be detected after antisense oligonucleotide treatment targeting the *Ube3a-*antisense transcript through at least 8 weeks post-treatment (*P* < 1e-15). Deviations in delta power from the expected natural history correlated with *Ube3a* expression in the mouse model (*P* < 0.001). Deviations in delta power from a human natural history model in Angelman syndrome can detect antisense oligonucleotide-mediated improvement in *Ube3a* expression in Angelman syndrome mice and may be relevant for human clinical trials.

## Graphical Abstract

**Graphical Abstract:**
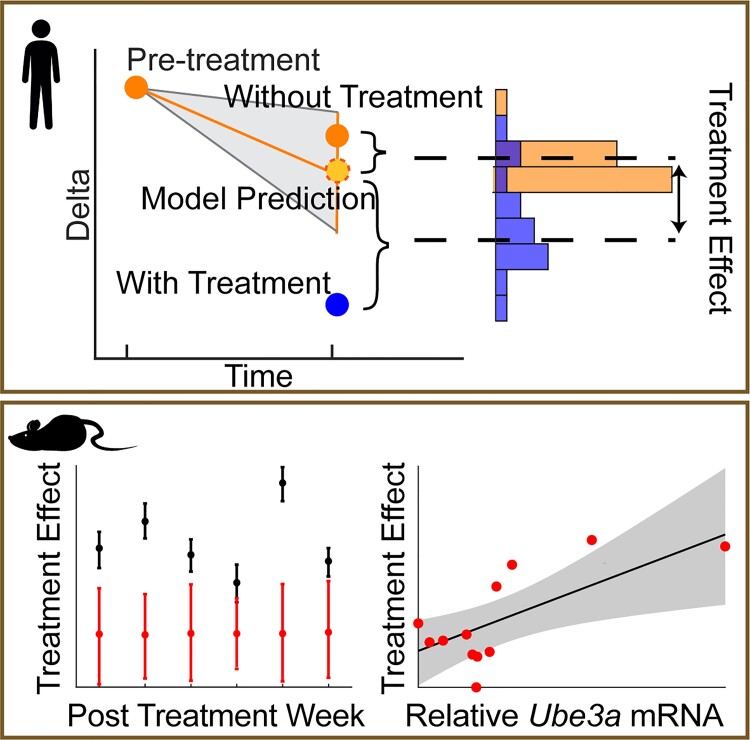

## Introduction

Angelman syndrome (AS) is a rare neurodevelopmental disorder^1–3^ characterized by severe developmental delay and epilepsy, along with impairments in speech and motor skills.^4,5^ AS is caused by a deficit of UBE3A protein due to genetic abnormalities resulting in loss of *UBE3A* expression from the maternal allele.^6,7^ Promising disease-modifying therapies to reinstate production of UBE3A are under development.^8^ In particular, antisense oligonucleotides (ASOs) have been developed to target the endogenous *UBE3A*-antisense transcript (*UBE3A-ATS*), which normally silences the paternal *UBE3A* allele in neurons.^9^ In a mouse model of AS, ASO treatment unsilences the paternal *Ube3a* allele and increases the production of UBE3A protein^9^ and has been shown to recover several associated phenotypes of the disease.^10^ Such potentially transformative disease-modifying treatments give rise to the need for an accurate, non-invasive approach to detect target engagement and treatment effect in clinical trials.

Many studies indicate that abnormal delta power (2–4 Hz) measured in the scalp EEG is a reliable and sensitive biomarker for AS. Delta power is highly elevated in AS subjects compared with typically developing individuals,^11–13^ and in mouse models of AS compared with wild-type (WT) mice.^11^ In addition, delta power correlates with genotype^12^ and cognitive function,^14,15^ where increased delta power correlates with more severely affected phenotypes. Therefore, delta power may present a useful biomarker for severity of disease and may provide a simple, non-invasive metric to track improvement in clinical trials. However, although delta power is reliably increased in AS compared with healthy control subjects, in cross-sectional studies, delta power measurements vary widely between individuals and throughout development. Longitudinal measurements of delta power, accounting for each subject’s age and elapsed time between measurements, are required to develop a more sensitive measure of target engagement in clinical trials.

Here, we first analyze a small database of prolonged continuous EEG recordings in AS subjects to show that delta power estimates remain stable over the course of a 24 h sample. Then, utilizing a large longitudinal data set of EEG recordings from subjects with AS, we develop a natural history model to predict delta power at a subsequent visit from delta power at an initial visit, age, and elapsed time between visits. We utilize this model to compute the sample and effect sizes needed to detect a treatment effect in a human clinical trial with 80% power. We then fit the model on a longitudinal AS mouse data set and measure for a treatment effect after ASO treatment. Finally, we compare deviations from the natural history model with *Ube3a* expression in *Ube3a-ATS* ASO-treated and control ASO-treated mice. This work provides a non-invasive method to detect potential treatment effects with confidence in AS and validates that increased *Ube3a* expression corresponds to deviations from the natural history of delta power in a mouse model of this disorder.

## Materials and methods

### Human subject data collection

Human subject data were obtained from (i) a database of EEG recordings from subjects with AS seen at Massachusetts General Hospital (MGH); and (ii) a database of EEG recordings from the multicenter AS Natural History Study (NHS; ClinicalTrials.gov identifier: NCT00296764) conducted as part of the Rare Diseases Clinical Research Network, Angelman, Rett and Prader-Willi syndrome consortium.

In (i), all AS subjects with longitudinal EEG recordings obtained between 2005 and 2019 were included. To prevent a disproportionate impact of subjects with multiple visits and to represent EEG recordings obtained across varying intervals of elapsed time between visits (i.e., inter-visit intervals or IVIs), pairings of longitudinal EEG recordings separated by hourly, daily, weekly, monthly, and yearly IVIs were included with an approximately equal distribution, as available for each subject. In total, 116 EEG recordings from 26 subjects (age: 0.89–32.5 years, 8 female:18 male, 2–13 visits per subject) were included. Recordings in this data set were separated by a median of 8.2 months (range: 2 days–4.4 years).

In (ii), subjects were recruited at six sites between 2006 and 2017 and EEG recordings were collected from the sites at Rady Children’s Hospital/University of California San Diego and Boston Children’s Hospital. Consent was obtained according to the Declaration of Helsinki and was approved by the institutional review boards of the participating sites. In total, 88 EEG recordings from 30 subjects (age: 1.3–21 years, 9 female:21 male, 2–6 visits per subject) were included. Recordings in this data set were separated by a median of 1.1 years (range: 8.4 months–7 years).

The final combined data set included 56 subjects with longitudinal EEG data from 204 total visits.

All EEG recordings were collected using the international 10–20 montage on either BioLogic or Xltek systems.^16^ For the MGH data, five recordings lasting ∼24 h were available. For the remainder of the data, recordings were of ∼50 min duration (mean 50 min, range: 3 min–6.5 hrs). The data were sampled at either 256 or 512 Hz, using a C2 spinous process reference. For the NHS data, 30 min of wake recording and 30 min of sleep recording were attempted at each session (mean: 28 min, range: 1 min–2.8 hrs). The data were sampled at 200–512 Hz, using a linked-ear reference. Some of the NHS subjects contributed EEG recordings that were obtained for clinical purposes.

All EEG data were manually staged for wake and sleep states by an experienced clinical neurophysiologist (CJC) and wake data selected for model development. We note that delta rhythms are abnormal during wake and sleep in AS.^11,14^ We focused on wake data because it was more widely available and more reliably identified. The final data set included wake recordings of mean duration 37.4 min (range: 1 min–6.5 hrs).

### Human EEG statistical analysis

Power spectra were calculated using the Chronux toolbox.^17^ Following the procedure in Ostrowski *et al.*,^14^ we analyzed only occipital and parietal electrodes, O1, O2, P3, Pz, and P4 referenced to a group average across those electrodes. There, it was shown that delta power estimates from the uncleaned posterior electrodes provided the best predictor of cognitive function as these electrodes are free of muscle artifacts and provide the largest amount of data from which to estimate delta power. For each channel, we computed the power spectrum on non-overlapping 1 s interval (1 Hz frequency resolution; Hanning taper). Within each interval, we estimated the relative delta power as the average 2–4 Hz summed power over channels divided by the 1–50 Hz total power over channels. We then averaged the delta power over all intervals to yield a single relative delta power value for each subject.

To examine the stability of relative delta power estimates across full day recordings, we analyzed EEG recordings from five subjects, each with at least 24 h of continuous recording and different genotypes (*n* = 2 deletion, *n* = 2 *UBE3A* mutation, *n* = 1 uniparental disomy). For this, we implemented a resampling procedure to estimate the relative delta power from 1 to 3600 randomly sampled (without replacement) 1 s epochs from the 24 h data set, reflecting data durations ranging from 1 s to 60 min. For each sample size (1 to 3600 s), and for each subject, we repeated this procedure 1000 times to compute the standard error of the mean (SEM) relative delta power estimate. For a given estimate of relative delta power, *δ*, we empirically estimate the 95% confidence interval from the resampling procedure. We repeated this same procedure using contiguous epochs of durations 1–60 min, choosing 1000 random start times in the resampling procedure.

For our natural history model, we fit a linear mixed effect model with dependent variable relative delta power at a subsequent time point, and fixed predictors of relative delta power at an initial time point, the interaction of log_10_(age) and IVIs, and a random intercept for subject. We declared a variable significant when the *P*-value of the *t*-test was <0.05. We also tested the significance of including genotype in the model by evaluating the Akaike information criterion. To validate our model, we employed a cross-validation procedure. For each iteration, the visits from one subject were left out for a test set and the model was trained on the remaining data. We compute the average root mean square error (RMSE) for the test set across all iterations.

To simulate treatment and control groups for power calculations, we sampled (with replacement) 25, 50, 100, or 150 subject EEG recording pairs for each group from the longitudinal data set. Then, from each subject in the simulated treatment group, we subtracted a fixed offset from the subject’s observed relative delta power at subsequent visit to represent a treatment effect under the hypothesis that treatment reduces delta power. We considered relative delta power offsets ranging from 0 to 0.1. For each fixed delta power offset, we repeated this simulation 2000 times and computed the proportion of times a difference between simulated treatment and simulated control groups was detected using a one-sided *t*-test with *P*-value < 0.05 (i.e., alpha = 0.05).

### Animals

All experiments were conducted in compliance with the rules set forth by the Biogen Institutional Animal Use and Care Committee in accordance with the guidelines established in the National Institutes of Health Guide for the Care and Use of Laboratory Animals. Mice were group housed on a 12 h light/dark cycle with ad libitum access to food and water. Both male and female offspring were used for experiments. Breeding was performed internally at Biogen by crossing female *Ube3a ^m+/p−^* (JAX Stock No: 016590) × male *Ube3a ^m+/p+^* (JAX Stock No: 000664) breeders to generate offspring, including experimental *Ube3a ^m−/p+^* (AS) mice and littermate WT *Ube3a ^m+/p+^* controls. WT and AS littermates were housed in the same cage whenever possible.

### Oligonucleotides

Synthesis and purification of all chemically modified oligonucleotides was performed as previously described.^18^ The 2′-MOE gapmer ASOs are 20 nucleotides in length, wherein the central gap segment comprising 10 2′-deoxynucleotides is flanked on the 5′ and 3′ wings by 5 2′-MOE modified nucleotides. The sequences of the ASOs are as follows: non-targeting control ASO, 5′-CTATAGGACTATCCAGGAA-3′ and mouse-specific *Ube3a-ATS* ASO, 5′-CCAGCCTTGTTGGATATCAT-3′.

### Antisense oligonucleotide *in vivo* administration

Lyophilized ASOs were dissolved in sterile PBS without calcium or magnesium and quantified by ultraviolet spectrometry. The ASOs were then diluted to the desired concentration required for dosing mice and sterilized through a 0.2 μm filter. Surgeries were performed +/− 3 days of postnatal Day 35 (P35). Mice were anaesthetized with 2% isoflurane and placed in a stereotaxic frame (David Kopf Instruments). After exposing the skull, a needle (Hamilton, 1701 RN 10 μl micro syringe, needle 26 s/2″/2) was used to penetrate the skull at 0.3 mm posterior and 1.0 mm lateral to the bregma and lowered to a depth of 2.25–3.0 mm (based on weight), to deliver a non-targeting control ASO or *Ube3a-ATS* ASO (500 μg) at a rate of ∼1 μl per 30 s into the cerebral ventricle. The needle was left in place for 5 min, slowly withdrawn and the incision was sutured.

### Sodium dodecyl sulfate–polyacrylamide gel electrophoresis (SDS-PAGE) western blotting

Mice were euthanized 8 weeks after intracerebroventricular (ICV) injection and the brains were rapidly harvested and cortical pinches of grey matter (200–300 mg) were flash-frozen. The tissues were homogenized and lysed in Pierce RIPA lysis and extraction buffer (Thermo Fisher Scientific, Waltham, MA) supplemented with 1% Halt protease and phosphatase inhibitor cocktail (Thermo Fisher Scientific, Waltham, MA) and centrifuged at 14 000*×g* for 20 min at 4°C to clear the lysate. Protein concentrations were determined using the Pierce BCA Protein Assay Kit (Thermo Fisher Scientific, Waltham, MA) and 10 μg of each sample was denatured in 6X SDS sample buffer (Boston Bioproducts, Ashland, MA) by boiling for 8 min at 90°C. Proteins were loaded into a Criterion 7.5% tris–glycine gel (Bio-Rad, Hercules, CA) and separated by SDS-PAGE at 120 V for 120 min. Gels were transferred to an IBlot2 nitrocellulose membrane (Invitrogen, Carlsbad, CA), blocked with TBST blocking buffer (Li-cor Biosciences, Lincoln, NE) for 1 h and washed three times with TBST. The membrane was probed with primary antibodies (1:1000 dilution) in antibody dilution buffer (1:1 TBST blocking buffer and 1X TBST) overnight at 4°C. The following antibodies were used for immunoblotting: UBE3A (1:1000; E8655, Sigma-Aldrich) and GAPDH (1:3000; 5174S, Cell Signaling Technology). After primary antibody staining, the blot was washed in triplicate with TBST and incubated for 1 h with secondary antibody (1:10 000 dilution of IR Dye 800 anti-mouse IgG and IR Dye 680 anti-rabbit IgG, Li-cor Biosciences, Lincoln, NE) in antibody dilution buffer. After a final triplicate wash with TBST, the blot was visualized using the Odyssey CLx imaging system (Li-cor Biosciences, Lincoln, NE).

### Quantitative reverse transcription polymerase chain reaction

Samples for polymerase chain reaction (PCR) were lysed in RLT buffer (Qiagen) + 0.1% β-mercaptoethanol and total RNA was extracted using the RNeasy mini kit (Qiagen) and RNase-free DNase set (Qiagen) following the manufacturers protocol. Total RNA concentration was determined using the Nanodrop 8000 spectrophotometer (Thermo Fisher Scientific, Waltham, MA) and reverse transcription was performed using the High Capacity cDNA Reverse Transcription kit (Applied Biosystems) using up to 2 µg of RNA as a template. About 50 ng of the resulting cDNA product was subjected to duplex PCR reactions using Gene Expression Master Mix (Applied Biosystems, Waltham, MA) containing Taqman primers for *Ube3a* (Cat# Mm00839910_m1), *Ube3a-ATS* (Cat# Mm02580988_m1) and housekeeping gene GAPDH (Cat# Mm99999915); all primers are from Applied Biosystems. Real-time PCR reactions were performed on the Via7 real-time PCR system (Thermo Fisher Scientific, Waltham, MA) using the following thermocycling conditions: 2 min at 50°C, 10 min at 95°C, followed by 50 cycles of 15 s at 95°C and 1 min at 60°C. Relative gene expression levels of *Ube3a* and *Ube3a-ATS* were calculated using the ddCt algorithm.

### Surgeries and local field potential recordings

Mice were surgically implanted with depth electrodes targeting layer IV of primary visual cortex 4–7 days after ASO administration (∼P42). Primary visual cortex was chosen to replicate and extend the delta phenotype observed previously in Sidorov *et al.* Mice were anaesthetized with 2% isoflurane and placed in a stereotaxic frame (David Kopf Instruments). A steel headpost was affixed to the skull anterior to bregma using cyanoacrylate glue. Burr holes (< 0.5 mm) were then drilled in the skull over binocular V1 (3.2 mm lateral of lambda). Tungsten electrodes (FHC, Bowdoinham, ME, US), 75 μm in diameter at their widest point, were implanted in each hemisphere, 450 μm below cortical surface. Reference electrodes consisted of 000–120 CS screws inserted into the skull touching dura located over prefrontal cortex. Wires extending from the electrodes were connected to female gold pins and inserted into a plastic pedestal connector (MS363 PlasticsOne, Roanoke, Va). Finally, dental cement (C&B Metabond, Parkell Inc., Bentwood, NY) was applied to form a stable, protective head cap. Recordings were performed using a Tucker-Davis Technologies R25D system at ∼3K sampling rate. Mice were head-fixed for all recording sessions viewing a full-field grey screen in an enclosed dark, quiet environment. Two weeks post-ICV infusion and 1 week post-surgery, the mice were habituated to this environment for two consecutive days, at 15 min per session. Recordings were then acquired during three consecutive daily 15 min sessions and results averaged for the ‘Week 2’ time point. A portion of mice were then recorded weekly thereafter.

### Mouse local field potential statistical analysis

Local field potential (LFP) analyses were performed with the experimenter blinded to treatment and genotype. Power spectra were calculated for each hemisphere using the Chronux software package^17^ in MATLAB (Mathworks) for the last 10 min of the ∼15 min recording session and averaged across hemispheres (5 tapers, time bandwidth product of 3, using 5 s windows with a 1 s overlap). A custom algorithm was used to remove recordings from channels with noisy LFPs, in some cases due to damage to the electrode. Absolute delta activity was measured as the peak activity between 1 and 5 Hz.

To examine model performance in the AS mouse model, we applied the same natural history model structure developed with the human data to the absolute delta power measurements from the longitudinal mouse LFP recordings. To estimate the model parameters for the mouse data, for each control ASO-treated mouse (*n* = 26), pairs of longitudinal recordings were defined using all possible combinations of subsequent recordings, resulting in 6–21 longitudinal pairs per mouse and 401 total longitudinal pairs.

To validate our model, we employed a cross-validation procedure similar to that used for the human data. For each iteration, the visits from one mouse were left out for a test set and the model was trained on the remaining data. Because the mouse data were collected at regular intervals, we also validated the model across weeks. For this, for each iteration, all the data from 1 week was left out for a test set, and the model was trained on the remaining data. We compute the average RMSE for the test sets across all iterations.

We then applied the natural history model to estimate a treatment effect in *Ube3a-ATS* ASO-treated versus control ASO-treated mice. To do so we compared the distribution of model residuals for the *Ube3a-ATS* ASO-treated and control ASO-treated mice. We note that because electrode implantation interferes with subsequent ICV injections (due to the required use of dental cement on the skull), we did not have pre-treatment absolute delta power values in the *Ube3a-ATS* ASO-treated mice. Therefore, to compute the model residual for an ASO-treated mouse, we randomly selected the absolute delta power value from a mouse in the youngest control ASO-treated group and used the ASO-treated mouse’s subject-specific variance to predict the absolute delta power value of the ASO-treated mouse at all subsequent ages. We repeated this procedure to compute the model residuals for each *Ube3a-ATS* ASO-treated mouse at all subsequent visits. We applied the same procedure to compute the model residuals for the control ASO-treated mice, while excluding pairing of the same mouse at both visits. To compute a treatment effect size for each postnatal week, we computed the difference in the median residuals of the *Ube3a-ATS* ASO-treated mice and the control ASO-treated mice. We repeated this entire procedure 10 000 times to generate a distribution of treatment effect sizes for each postnatal week. We used this distribution to compute the 95% confidence bounds, extending from the 2.5% to 97.5% quantile of the distribution. We note that inter-subject variability is taken into account by utilizing all mouse data from each week.

To test the null hypothesis of no difference in median residuals between *Ube3a-ATS* ASO-treated and control ASO-treated mice, we implemented a resampling procedure. Under the null hypothesis of no difference between groups, we first created a combined group including both the control ASO-treated (*n* = 26) mice and *Ube3a-ATS* ASO-treated (*n* = 15) mice at post-treatment Week 2 (∼postnatal Day 49). Then, from this list, we selected randomly (without replacement) two pseudo-groups of mice to represent pseudo-control ASO-treated (*n* = 26) and pseudo-ASO-treated (*n* = 15). We repeated this entire procedure 10,000 times to create distributions of treatment effect size for each week, assuming no difference between groups. To test our hypothesis that *Ube3a-ATS* ASO-treated mice would have larger positive residuals compared to control ASO-treated mice, we compared the observed and resampled distributions of treatment effect size at each week using a one-sided *t*-test with significance at *P* < 0.05.

### Statistical analysis for *Ube3a* mRNA upregulation and model residuals

To assess the correlation between the relative *Ube3a* mRNA upregulation and the model residuals, we utilized the natural history model to compute residuals for mice at Week 7 and compare them to measured *Ube3a* mRNA levels obtained in the same mice at Week 8, in both control ASO-treated (*n* = 8) and *Ube3a-ATS* ASO-treated (*n* = 4) mice. Only a portion of the mice were sacrificed at the Week 8 time point for mRNA analysis as other experiments were run using the remaining mice. No mice were lost prematurely. To compute model residuals, for each Week 7 mouse, we compared the predicted and observed absolute delta values using a randomly selected control ASO-treated mouse at Week 2. We then matched this residual with the *Ube3a* mRNA measurements from the same mouse. We repeated this procedure for all 12 mice with *Ube3a* mRNA measurements at Week 8. We then performed linear regression to estimate the slope from a data set of 12 samples. We repeated this entire procedure 1000 times (each using randomly selected control ASO-treated mouse at Week 2) to create a distribution of slope estimates.

### Data availability

Derived human subject data obtained from MGH are available upon reasonable request from the corresponding author. Human subject data from the NHS are available at ClinicalTrials.gov (NCT00296764). Derived mouse data are available upon reasonable request from corresponding author.

## Results

### Delta power estimates are stable over a 24 h period within an individual

**Figure 1:**
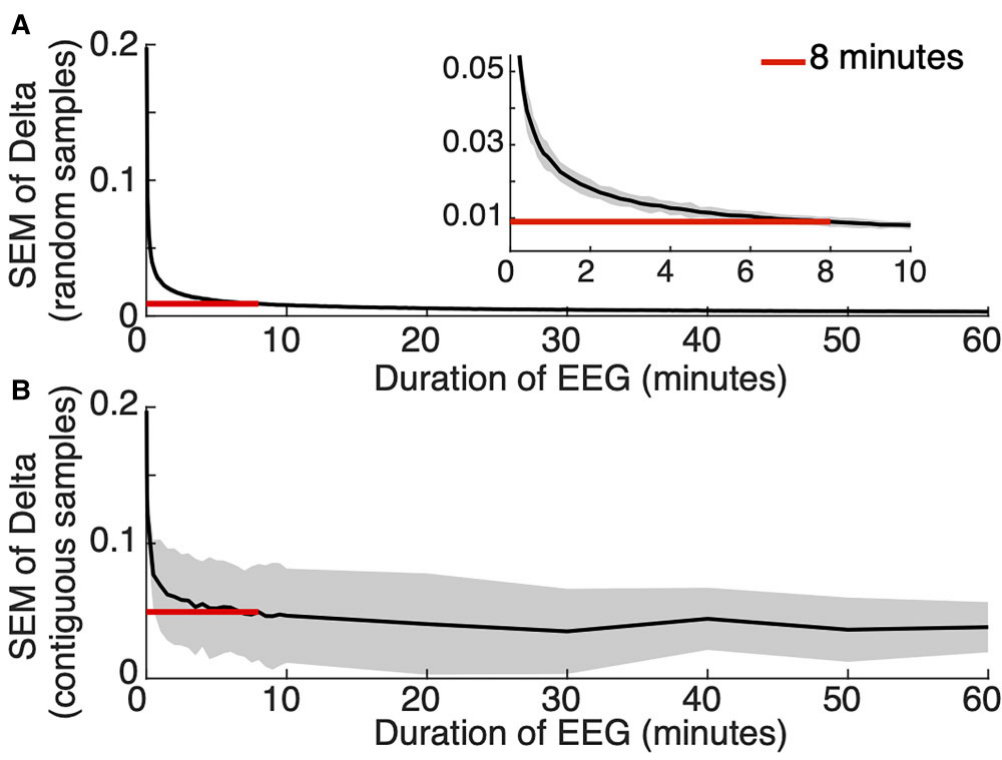
**Only a few minutes of EEG data are needed to estimate delta power with high precision.** The black curved line represents the SEM in the estimate of relative delta power calculated on a given amount of data (**A**) random 1 s samples or (**B**) contiguous samples and shaded bars represent 95% confidence. Red straight line indicates 8 min of data.

Spectral estimates from scalp EEG are highly variable within individuals over the course of years.^12,14^ To assess the stability of relative delta power estimates over shorter time intervals, we compared relative delta power estimates using increasing data sample sizes, randomly selected from 1 s interval in five AS subjects with 24 h continuous recordings (see Materials and methods). As the amount of data increases, the standard error decreases at a rate of ∼$1/\sqrt{x}$ (Fig. 1A). When the sample size exceeds 8 min, the mean SEM plateaus and remains <0.009 [95% CI (0.008, 0.01)]. We repeated this analysis using contiguous intervals of data (Fig. 1B) and found consistent results; when the sample size exceeds 8 min, the mean SEM plateaus and remains less than 0.05 [95% CI (0.015, 0.08)], although with a larger variability in the estimate due to expected daily variations in relative delta power. Therefore, we conclude that relative delta power estimates are stable over the course of a 24 h period and can be reliably estimated from just 8 min of EEG data.

### Delta power at a future visit can be reliably predicted from a longitudinal natural history model

**Figure 2:**
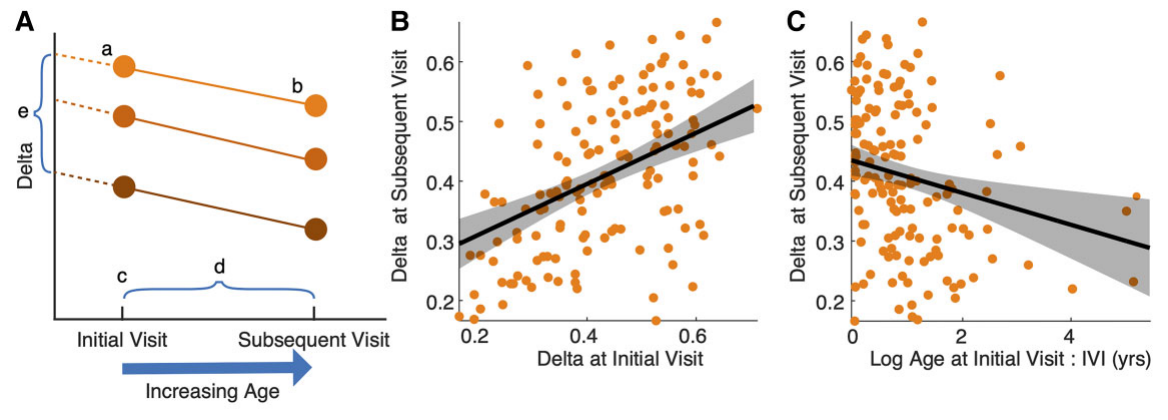
**Natural history model overview.** (**A**) Schematic of variables included in the natural history model: (a) delta power at initial visit (unitless); (b) delta power at subsequent visit (unitless); (c) age at initial visit; (d) IVI; (e) random intercept. (**B, C**) Model fit on longitudinal data. Each dot indicates the relative delta power measurements for each subject and visit. The solid line indicates the model fit using mean values for the other two predictors. The shaded regions indicate 95% confidence intervals. (**B**) Relative delta power at initial visit (unitless) positively correlates with relative delta power at subsequent visit (unitless). (**C**) The interaction of log_10_(age) at initial visit and IVI negatively correlates with relative delta power at subsequent visit.

We use the human longitudinal data set to estimate parameters in a natural history model of delta power in AS to predict relative delta power at a subsequent visit. To do so, we constructed a linear regression model with three predictors: relative delta power at the initial visit, log_10_(age) at the initial visit,^11,19^ and elapsed time between the visits (IVI). Because the data consist of multiple longitudinal observations from repeat subjects, we include a random intercept to allow inter-subject variability in baseline delta power, for example due to genotype.^12^ The final natural history model is (Fig. 2A):

$$Delta_{Subsequent\;Visit}\;∼\;Delta_{Intial\;Visit}+log_{10}(Age_{Initial\;Visit}\;):IVI+(1|Subject).$$

**Figure 3:**
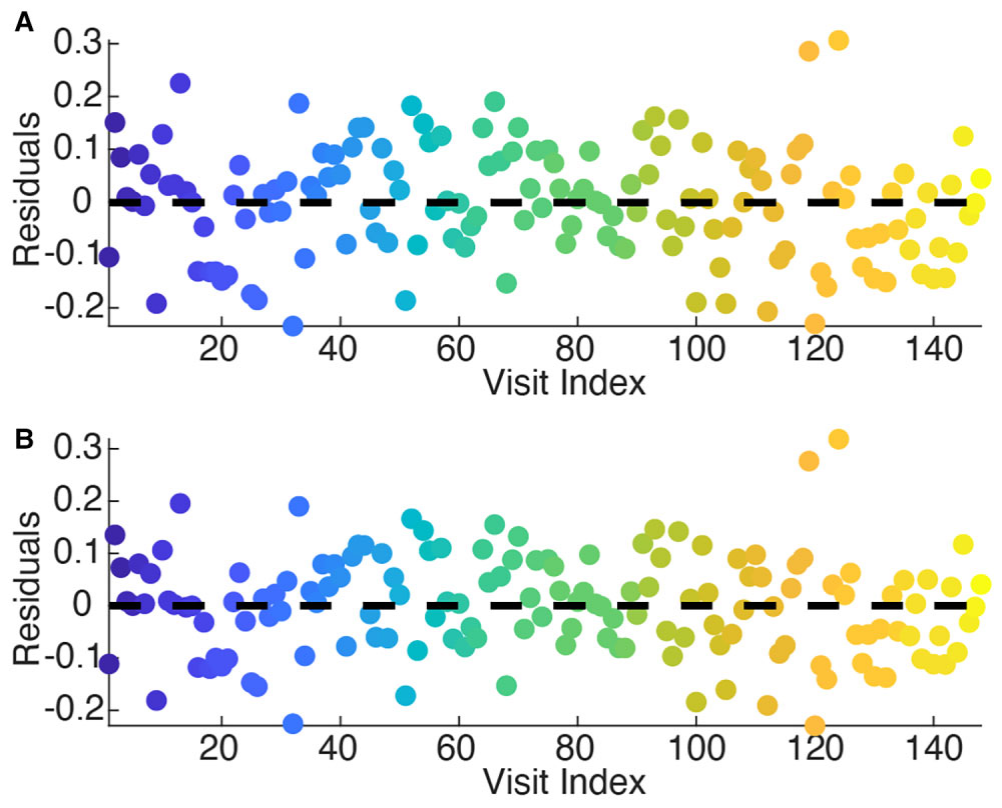
**Leave-one-out cross-validation.** The model residuals are plotted for (**A**) leave-one-out cross-validation, where for each iteration, the visits from one subject were left out for a test set and the model was trained on the remaining data, and (**B**) the full model. (**A, B**) The residuals are uniform across subjects in both cases. Each colour represents the residuals from the same subject.

Fitting this model, we find that each predictor is significant. Relative delta power at the initial visit is the strongest predictor; for every unit increase in relative delta power at the initial visit, there is a 0.43 unit increase in relative delta power at the subsequent visit [95% CI (0.28, 0.57), *P* < 1e-7; Fig. 2B]. The interaction of log_10_(age) and IVI negatively correlates with relative delta power at the subsequent visit [effect size −0.03, 95% CI: (−0.04, −0.009), *P* = 0.003; Fig. 2C and Table 1], where older ages and longer IVIs correlate with reduced delta power at the subsequent visit. We note that including genotype in the model did not improve model fit (effect size 0.03, *P* = 0.23). We find no evidence that the residuals differ by genotype (two-sided *t*-test, *P* = 0.28). To validate our model, we employ a leave-one-out validation (Fig. 3). We find uniform residuals and a consistent RMSE (0.09 for the leave-one-out model versus 0.1 for the full model) indicating generalizable fit.

**Table 1:** Model fit for human data

	Parameter estimate	95% Confidence bounds	*P*-value
Intercept	0.25 unitless	(0.18,0.32)	<1e-10
Delta initial visit	0.43 unitless	(0.28,0.57)	<1e-7
Log_10_(Age_Initial Visit_):IVI	−0.03 years	(−0.04,-0.009)	0.003

### Power to detect deviation from the natural history model following treatment

**Figure 4:**
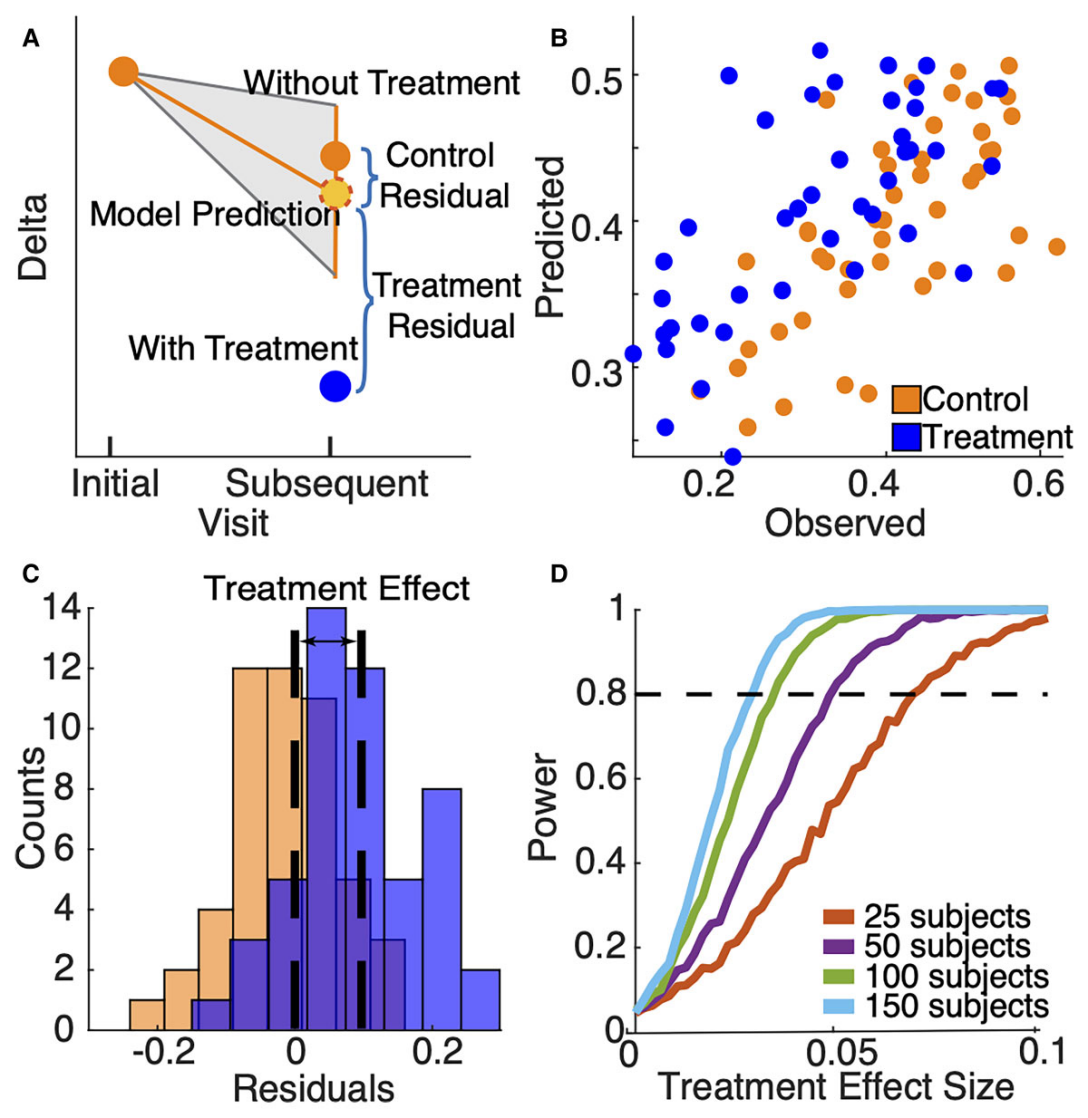
**Simulation of model implementation.** (**A**) Illustration of the longitudinal model to detect a treatment effect. If delta power is reduced post-treatment, the model residuals for the treatment group will be be larger than for the control group. Example simulation (**B, C**) where the effect size of the treatment group is a 0.1 reduction in delta power. (**B**) Predicted values from the model versus the observed values of delta power at a subsequent visit for the control (orange) and treatment simulated data (blue). (**C**) Histograms of the corresponding control and treatment residuals. Black dashed lines indicate mean residuals of each group. Black arrow indicates the difference between mean residuals of each group, i.e., the treatment effect. (**D**) Power to detect a treatment effect versus treatment effect size when sampling 25, 50, 100 and 150 subjects per group. Black dashed line indicates 80% power.

An accurate natural history model can be used to identify a deviation from the expected natural history due to treatment, i.e., a treatment effect. Delta power is abnormally increased in AS^11–13^ and higher delta power correlates with more severe disease;^12,14,15^ therefore, we expect effective treatment to reduce delta power (illustration in Fig. 4A). To determine if relative delta power is significantly reduced beyond the expected natural variability for an individual subject, the model-predicted relative delta power can be compared with the observed delta power at the subsequent visit. To test the hypothesis that our model can detect treatment effects, i.e., reduced relative delta power beyond its natural variability, we compare the residuals, or deviations from the model, of the simulated control and treatment groups. We expect a treatment effect that reduces relative delta power would result in significantly larger model residuals in the simulated treatment group compared to the simulated control group (Fig. 4A).

To determine the sensitivity of the natural history model to detect a difference between groups, we simulate power calculations based on varying sample sizes and treatment effect sizes. To illustrate this simulation procedure, we consider a theoretical control group (*n* = 50 subjects) and a simulated treatment group (*n* = 50 subjects, see Materials and methods). For the treatment group, we simulate varying treatment effects and sample sizes. For example, if we assume that treatment results in a 0.1 decrease in the observed relative delta power, using the longitudinal natural history model, we find larger residuals in the treatment group compared to the control group in this example, as expected [effect size 0.11, 95% CI: (0.08, Inf), *P <* 1e-8, one-sided *t*-test, Fig. 4B, C]. Repeating this simulation for different effect and sample sizes (Fig. 4D), we conclude we can detect with 80% power a treatment effect size of 0.064 relative delta power in a sample of 25 subjects per group, 0.046 in a sample of 50 subjects per group, 0.033 for 100 subjects per group and 0.027 for 150 subjects per group.

We note that this procedure, applied to our natural history model, could serve as the control data set for a future clinical trial. In that case, the number of untreated control subjects in the model would be sampled to match the number of enrolled and treated subjects.

### The natural history model of delta activity detects deviations in delta power in antisense oligonucleotide-treated Angelman syndrome mice

**Figure 5:**
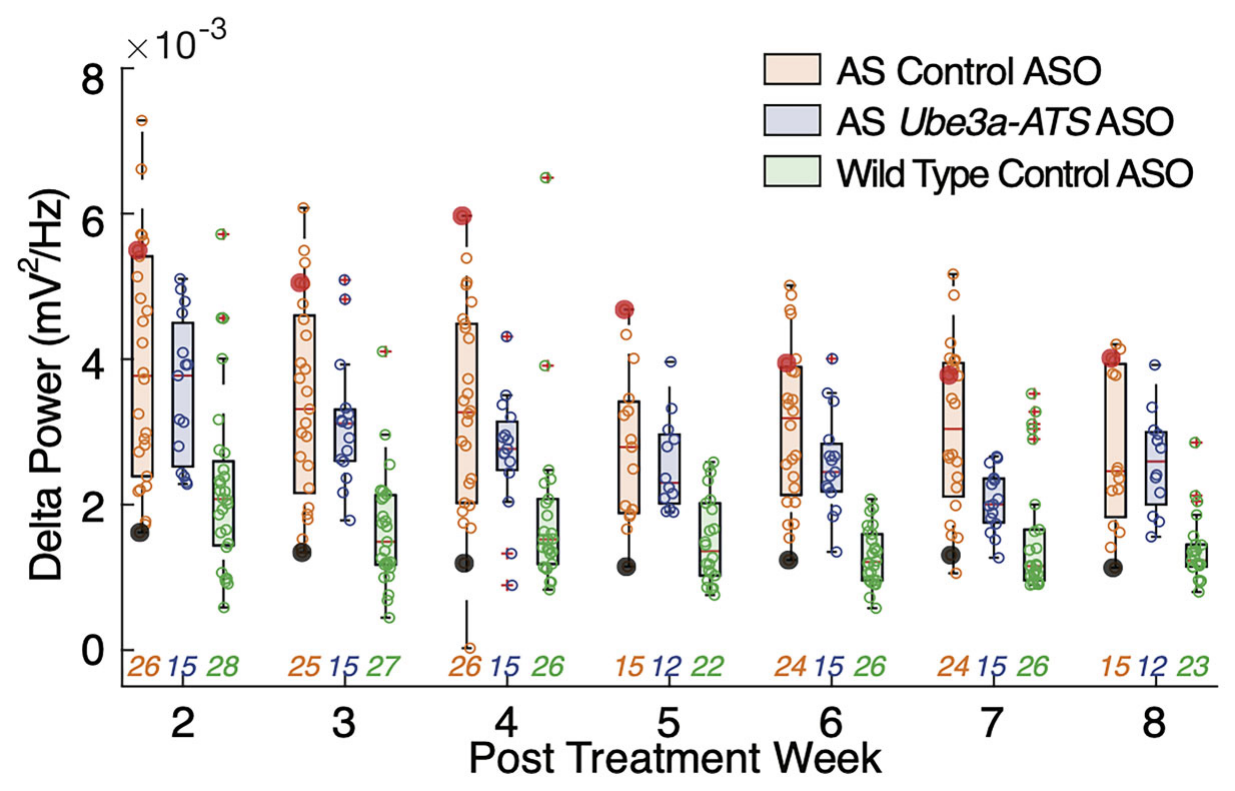
**Longitudinal mouse LFP data.** Boxplots of delta power across age per group at each time point (minimum, maximum, and median indicated): mice with AS that received control ASO treatment (orange, left boxplot), mice with AS that received *Ube3a-ATS* ASO treatment (blue, middle boxplot), and wild-type mice that received control ASO treatment (green, right boxplot). The filled marker with different colour (red and black) each represents the progression of an example control ASO-treated mouse across weeks. The median of each group is indicated by a horizontal line. Using cross-sectional measures, no difference in delta power can be detected between control-treated AS mice and *Ube3a-ATS* ASO-treated AS mice at any week (*P >* 0.05) except Week 7 (*P* = 0.0016). Control-treated animals have higher delta values than WT at each week (*P <* 0.01). ASO-treated animals have higher delta values than WT at each week (*P <* 0.01), except Week 4 (*P* = 0.2). The numbers of animals per group for each week are indicated below each boxplot, with corresponding group colours.

To determine whether the *Ube3a-ATS* ASO was able to correct abnormal delta-like (1–5 Hz) oscillations, AS mice were injected with a single ICV infusion of either the non-targeting control ASO or the *Ube3a-ATS* ASO (500 ug) at P35. WT mice only were injected with the control ASO. LFP recordings in the visual cortex were obtained weekly from 2 weeks through 8 weeks post-ICV infusion [WT control ASO (*n* = 28), AS control ASO (*n* = 26), AS *Ube3a-ATS* ASO (*n* = 15)]. On a visual inspection, absolute delta power in AS control-treated ASO mouse models decrease with age, consistent with prior reports^11^ (Fig. 5 orange). We find a main effect of both age and ASO treatment when including all weeks (two-way ANOVA; treatment, *P* = 0.003; age, *P* < 1e-4). When evaluating each week directly, we did not detect a significant difference between AS *Ube3a-ATS* ASO mice and AS control ASO mice for any post-treatment week (one way ANOVA, *P >* 0.15) except at Week 7 (*P* = 0.003, Fig. 5).

To assess the ability of the natural history model to detect treatment effects in the mouse data, we first estimated model parameters on all available longitudinal data from the AS mice treated with control ASO. As described in the Methods, since no pre-treatment observations of delta power were available for the mice, randomly selected Week 2 visits from the control group were used instead. Doing so, we found trends in all three predictors consistent with analysis of the human data; absolute delta power at a subsequent recording increased with delta power at an initial recording [effect size 0.06, 95% CI: (−0.01, 0.14), *P* = 0.1; Table 2], and decreased with the interaction of log_10_(age) and IVI [effect size −8.07e-5, 95% CI: (−1.58e-4, −3.01e-6), *P* = 0.04].

**Table 2:** Model fit for mouse data

	Parameter estimate	95% Confidence bounds	*P*-value
Intercept	3.08e-3 mV^2^/Hz	(2.58e-3, 3.59e-3)	<1e-10
Delta initial visit	0.06 mV^2^/Hz	(−0.01, 0.14)	0.10
Log_10_(Age_Initial Visit_):IVI	−8.07e-5 weeks	(−1.58e-4, −3.01e-6)	0.04

**Figure 6:**
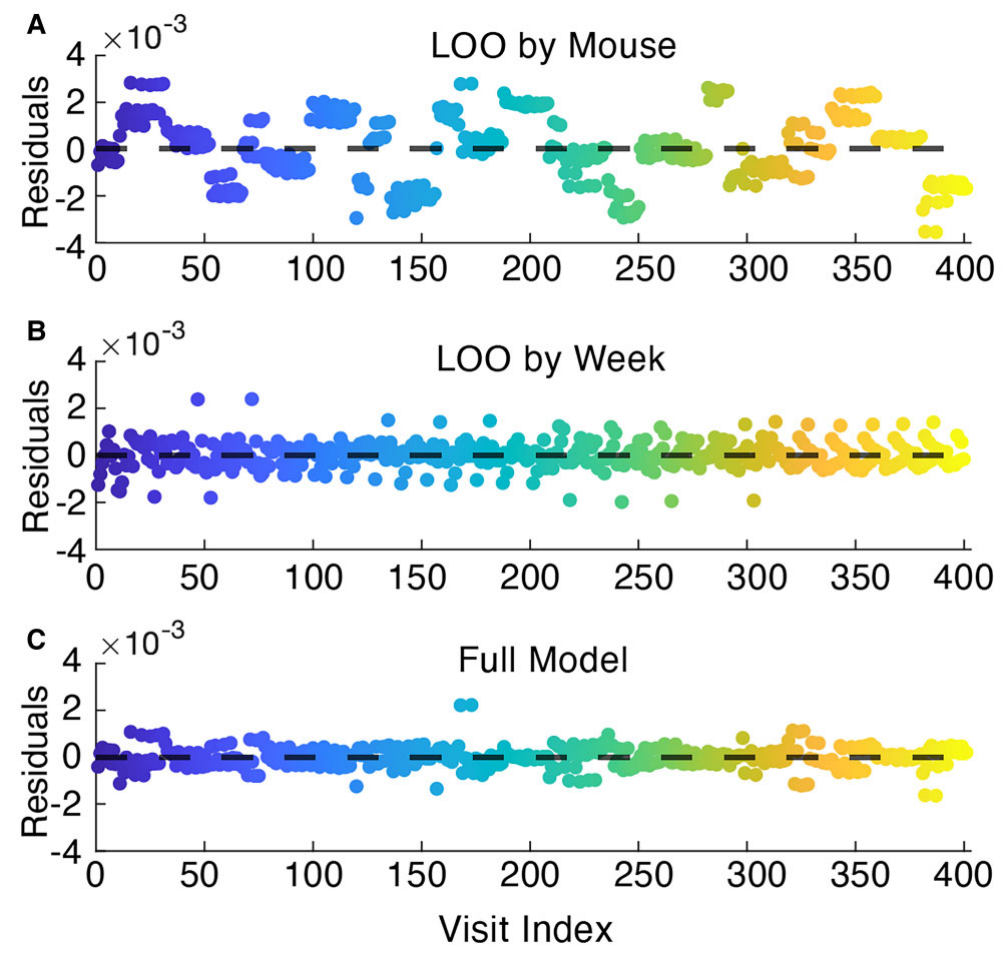
**Leave-one-out cross-validation for the rodent model.** The model residuals (mV^2^/Hz) are plotted for (**A, B)** leave-one-out cross-validation by (**A**) mouse and (**B**) week, where for each iteration, the recordings from one mouse or from 1 week were left out for a test set and trained on the remaining data. (**C)** The model residuals (mV^2^/Hz) for the full model. In each plot, the horizontal dashed line is the median residual.

To validate our model on the rodent data, since the data was collected at uniform time points, we employed two cross-validation strategies, leave-one-out by mouse and leave-one-out by week (see Materials and methods). Using both strategies, we find uniform residuals, and a consistent RMSE: 0.0014 mV^2^/Hz for the leave-one-out model by mouse, 5.8e-4 mV^2^/Hz for the leave-one-out by week and 4.5e-4 mV^2^/Hz for the full model (Fig. 6).

**Figure 7:**
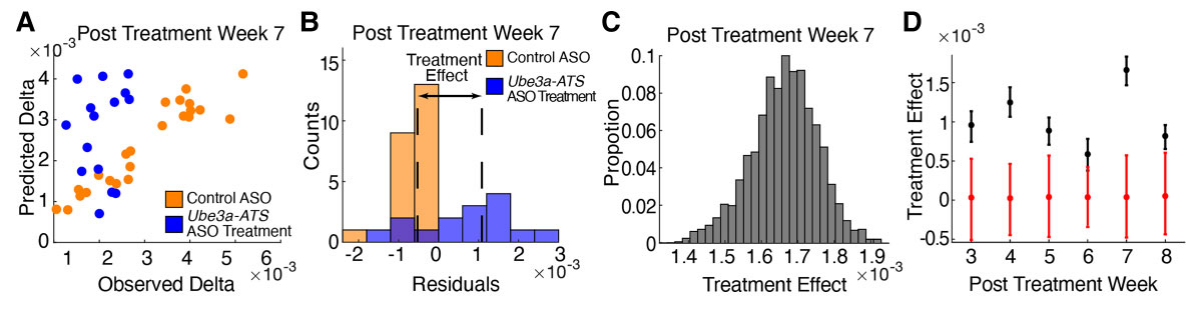
**Application of natural history model to mouse model of AS.** (**A**) Example of predicted versus observed delta power (mV^2^/Hz) values at Visit 2 from one prediction iteration of the resampling procedure corresponding to Week 7 post-treatment after *Ube3a-ATS* ASO (blue) or control ASO (orange) treatment. (**B**) Corresponding example histogram of *Ube3a-ATS* ASO treatment and control ASO residuals from the same iteration. Dashed lines indicate the median residual of each group. The distance between the dashed lines is the treatment effect (mV^2^/Hz). (**C**) Histogram of estimated treatment effect at Week 7 post-treatment from 10 000 iterations. (**D**) Treatment effect size versus post-treatment week. Black circles (error bars) are mean (95% confidence bounds) of treatment effect at each age. Red circles (error bars) are the treatment effect between two groups under null hypothesis. We note that Week 2 observations serve as the initial visit.

To assess the impact of treatment, we compared model residuals for control ASO-treated and *Ube3a-ATS* ASO-treated mice using a resampling procedure (see Materials and methods; Fig. 7A). We computed the treatment effect (i.e., median residual difference; Fig. 7B) between the control ASO-treated and *Ube3a-ATS* ASO-treated mice for each age following treatment (example in Fig. 7C) and compared with a null distribution (Fig. 7D). We found a significant treatment effect for all ages lasting through the 8 weeks observed, with the strongest treatment effect present at age Week 7, corresponding to 7 weeks after treatment. We note that treated and untreated subjects are not required to have the same ages and IVIs using the natural history model. For example, the natural history model is able to detect a treatment effect between Week 4 ASO-treated mice and Week 8 control ASO-treated mice (*P* < 0.05 for all simulations), while a longitudinal paired *t*-test cannot (*P* < 0.05 for 1.3% of simulations). This is likely due to the impact of older ages and IVIs, resulting in relatively lower delta power, in the control group. We conclude that the natural history model can detect a significant and long-lasting treatment effect in *Ube3a-ATS* ASO-treated mice compared to control ASO-treated mice across multiple ages and IVIs.

### Unsilencing of the *Ube3a* paternal allele with a *Ube3a-ATS* antisense oligonucleotide in Angelman syndrome mice

**Figure 8:**
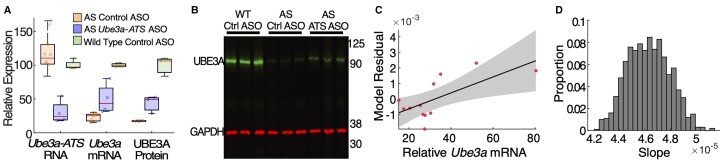
**Unsilencing of the *Ube3a* paternal allele with a *Ube3a-ATS* ASO in AS mice leads to increase *Ube3a* mRNA and UBE3A protein and correlation to model residuals.** (**A**) *Ube3a-ATS* RNA levels (normalized to WT control) after treatment with a non-targeting control ASO (orange) or a *Ube3a-ATS* ASO in WT or AS mice. Mice were ICV dosed at P35 and cortical tissue was collected at 8 weeks post-ASO treatment. (WT control ASO = 100 ± 3%, *n* = 4; AS control ASO = 113 ± 8%, *n* = 8; AS *Ube3a-ATS* ASO = 30 ± 8%, *n* = 4) (left). *Ube3a* mRNA levels (normalized to WT control) after control or *Ube3a-ATS* ASO treatment (WT control ASO = 100 ± 1%, *n* = 4; AS control ASO = 22 ± 2%, *n* = 8; AS *Ube3a-ATS* ASO = 50 ± 11%, *n* = 4) (middle). UBE3A protein levels. UBE3A signal intensity was quantified relative to GAPDH (WT control ASO = 100 ± 8%, *n* = 3; AS control ASO = 17 ± 1%, *n* = 3; AS *Ube3a-ATS* ASO = 44 ± 8%, *n* = 3) (right). Each group (*Ube3a* mRNA, *Ube3a-ATS* RNA and UBE3A protein) was normalized to its own respective WT control group. Error bars are SEM and *n* = number of mice; Two-way ANOVA with Tukey’s multiple comparisons test, *P < 0.05, **P < 0.01, ***P < 0.001, ns = not significant. (**B**) Western blot from WT and AS mouse cortical tissue. The uncropped blot is available in Supplementary Figure 1. (**C**) Example model residuals from Week 7 mice (red dots, mV^2^/Hz) and *Ube3a* mRNA expression (normalized to WT control, unitless) and the linear fit to these data (mean, black line; grey-shaded region, 95% confidence intervals). (**D**) Histogram of estimated slopes from all resamples.

To assess *Ube3a* unsilencing in vivo, we compared the levels of *Ube3a-ATS* and *Ube3a* mRNA by qPCR in cortical tissue 8 weeks after treatment with control ASO or *Ube3a-ATS* ASO (500 ug) in WT (*n* = 4 control ASO) or AS mice (*n* = 10 control ASO; *n* = 4 *Ube3a-ATS* ASO). We observed a ∼70% reduction in *Ube3a-ATS* with the *Ube3a-ATS* ASO compared with control ASO-treated mice at 8 weeks post-ASO administration. In a subset of mice for whom the data were available, this level of *Ube3a-ATS* knockdown corresponded to ∼2-fold increase in *Ube3a* mRNA levels compared with AS control ASO-treated mice and levels that were ∼50% of WT control ASO-treated mice (Fig. 8A). To understand the correlation between *Ube3a* mRNA and protein levels, we next quantified (by western blot) UBE3A protein levels in cortex in a subset of mice used for the RNA quantification at 8 weeks after ASO administration. After *Ube3a-ATS* ASO administration, UBE3A protein levels were 44% of WT control ASO-treated mice (∼2-fold increase compared with the AS control ASO group) 8 weeks post-ASO (Fig. 8B; see Supplementary Fig. 1 for full uncropped western blot). We conclude that *Ube3a-ATS* ASO ICV infusion successfully increases cortical UBE3A protein levels in AS mice.

### Increased model residuals correlate with increased *Ube3a* mRNA production

To assess correlation between the relative *Ube3a* levels and the model residuals, we utilized the direct measures of *Ube3a* mRNA available for *Ube3a-ATS* ASO-treated (*n* = 4) and control ASO-treated mice (*n* = 8). We then implemented a resampling procedure (controlling for the small sample size, see Materials and methods) to estimate the slope relating the model residuals and the relative *Ube3a* mRNA levels. We found a positive relationship between relative *Ube3a* mRNA and the model residuals [mean (std) slope = 4.63e-5 (1.66e-6), standard error = 5.26e-8], all fitted slopes are positive with *P <* 0.001, (Fig. 8C, D). We conclude that larger deviations from the natural history of absolute delta power correlate with increased relative *Ube3a* expression.

## Discussion

As potentially disease-modifying therapies are on the horizon for AS subjects, reliable methods to measure and detect treatment response are needed. Here, we utilized a large database of longitudinal EEG recordings from AS subjects and to develop a natural history model of relative delta power in this disorder. As demonstrated in simulation, the model allows estimation of the populations required to detect treatment effects of various sizes on relative delta power for use in clinical trial planning. We also validated the therapeutic utility of this model in showing that it can detect deviations in absolute delta power in a mouse model of AS following *Ube3a-ATS* ASO treatment compared with mice treated with a control ASO. These results support utilizing non-invasive measures of delta power to demonstrate target engagement and potential treatment effect in human clinical trials in AS.

Abnormally increased delta power is a consistent electrophysiologic phenotype of AS across species that shows promise to provide a meaningful biomarker for treatment efficacy. One challenge in employing this biomarker in clinical trials is that delta power varies dramatically across subjects and with age.^12,14^ Here, we showed that stable estimates of relative delta power can be obtained from <10 min of EEG data. Leveraging the advantages of a longitudinal mixed effects model over conventional statistical approaches,^20^ we then used a large longitudinal human data set to develop a natural history model of relative delta power and showed that an individual’s future delta power values could be predicted using readily available non-invasive clinical data (i.e., delta power at an initial visit, and the interaction of age and IVI). By including the prior delta power estimates and a random intercept, the longitudinal model controls for specific variations in baseline delta power due to genotype and disease severity and reduces noise due to inter-subject variability. Noise is further reduced in our longitudinal model by accounting for the known impact of IVI and interaction of age:genotype on delta power. This model then can be used to identify deviations in delta power outside of the expected natural history with confidence and thereby identify a significant treatment effect.

Using the natural history model, we performed power calculations to characterize the effect sizes and sample sizes needed to identify a treatment effect. We showed by simulation that in a sample of 50 treated and 50 untreated AS subjects, a decrease in relative delta power of 0.046 could be detected with 80% confidence, smaller than the reported 0.2 difference in relative delta power observed between controls and AS children^11^ and consistent with an increase in the raw score of the cognitive domain of the Bayley Scales of Infant and Toddler Development, 3rd edition^14^ of only 0.6 points. We conclude that small deviations in relative delta power, potentially signalling a treatment effect, can be detected by the model, even when below the threshold of detection on a performance-based test. We note that we could have used absolute or relative delta power for our analysis; however, we chose relative delta power motivated by previous work^14^ and to relate these changes to cognitive function. We note that changes in relative delta power can occur due to changes in absolute power at higher frequencies. However, we expect the impact of such changes to be small due to the rapid decrease in power with frequency.

Several groups have reported promising approaches to reinstate *Ube3a* expression by unsilencing the paternal allele using an ASO treatment in mouse models of AS.^9,10^ Similar to these reports, we found that *Ube3a-ATS* ASO treatment correlated with increased *Ube3a* expression. Using our natural history model of absolute delta power, we were able to both detect a persistent treatment effect following *Ube3a-ATS* ASO treatment and found that the changes in absolute delta power correlated with *Ube3a* expression. These differences were not reliably detected using direct comparisons, but by controlling for variations expected with age, IVI, and inter-subject variability, the longitudinal natural history model had the power to detect a significant treatment effect between the treated and control groups, beyond changes expected with the natural history. The mice data analyzed here were limited by requiring us to implement model predictions between different individual mice, as in a cross-sectional data set. If longitudinal data were available, we would expect the model to be even more sensitive to detect a treatment effect between groups. The mice data were also limited by a small sample size for those with both neurophysiological measurements and *Ube3a* mRNA expression estimates. Additional data would facilitate a more direct correlation between deviations from the natural history model and mRNA expression within the *Ube3a-ATS* ASO treatment group. Finally, we did not test a vehicle control AS group in our data, though we note that prior studies have already demonstrated a non-targeting ASO has similar mRNA to AS.^21^ In addition, *Ube3a-ATS* ASO treatment has been shown to result in a sustained increase in *Ube3a* expression up to 4 months after treatment, corresponding to improved synaptic plasticity and cognitive functions.^9^ The longitudinal model developed herein enables testing for and detection of a treatment effect at any IVI post-treatment, thus enabling detection of peak effects and duration. Future work evaluating the natural history of delta power across the entire lifespan in AS mouse models, including early development, may be helpful to elucidate the impact and duration of *Ube3a-ATS* ASO treatment at different ages.

With many potentially disease-modifying treatments for AS in development, we introduce a natural history model and statistical procedure to utilize deviations from expected measurements of delta power as a sensitive indicator of target engagement and possible treatment efficacy. Measures of *Ube3a* expression in mouse models can be done by directly assaying neuronal tissue. Estimates of UBE3A neuronal levels in humans may be performed indirectly through cerebrospinal fluid (CSF) sampling using lumbar puncture. A reliable EEG biomarker to complement CSF data would reduce the need for invasive procedures and would enable multiple repeated measures for longitudinal observations. We find that delta power provides a simple, non-invasive alternative to invasive UBE3A measurements. Further, as enrolment of subjects with rare neurological diseases like AS in clinical trials can be challenging, our natural history model allows enrolment of subjects at different ages and follow-up intervals, and may obviate the need to enrol a control group. Future work to validate the relationship between delta power and UBE3A expression after effective treatment in humans with AS would secure delta power as a mechanistic biomarker to gauge both target engagement and therapeutic response in clinical trials.

## Supplementary Material

fcac106_Supplementary_Data

## Abbreviations

AS =: Angelman syndrome
ASO =: antisense oligonucleotide
CSF =: cerebrospinal fluid
IVI =: inter-visit interval
ICV =: intracerebroventricular
LFP =: local field potential
MGH =: Massachusetts General Hospital
NHS =: Natural History Study
P =: postnatal day
RMSE =: root mean square error
SDS-PAGE =: sodium dodecyl sulfate–polyacrylamide gel electrophoresis
SEM =: standard error of the mean
*Ube3a-ATS* =: *Ube3a*-antisense transcript
WT =: wild-type

## References

[1] Petersen MB , Brøndum-Nielsen K, Hansen LK, Wulff K. Clinical, cytogenetic, and molecular diagnosis of Angelman syndrome: Estimated prevalence rate in a Danish county. Am J Med Genet. 1995;60:261–262. 7573182 10.1002/ajmg.1320600317

[2] Kyllerman M . On the prevalence of Angelman syndrome. Am J Med Genet. 1995;59:405. 8599374 10.1002/ajmg.1320590331

[3] Mertz LGB , Christensen R, Vogel I, et al Angelman syndrome in Denmark. Birth incidence, genetic findings, and age at diagnosis. Am J Med Genet Part A. 2013;161:2197–2203. 10.1002/ajmg.a.36058 23913711

[4] Williams CA , Beaudet AL, Clayton-Smith J, et al Angelman syndrome 2005: Updated consensus for diagnostic criteria. Am J Med Genet. 2006;140A:413–418. 10.1002/ajmg.a.31074 16470747

[5] Thibert RL , Larson AM, Hsieh DT, Raby AR, Thiele EA. Neurologic manifestations of Angelman syndrome. Pediatr Neurol. 2013;48:271–279. 23498559 10.1016/j.pediatrneurol.2012.09.015

[6] Bird LM . Angelman syndrome: Review of clinical and molecular aspects. Appl Clin Genet. 2014;7:93–104. 24876791 10.2147/TACG.S57386 PMC4036146

[7] Sonzogni M , Hakonen J, Bernabé Kleijn M, et al Delayed loss of UBE3A reduces the expression of Angelman syndrome-associated phenotypes. Mol Autism. 2019;10:1–9. 31143434 10.1186/s13229-019-0277-1 PMC6532248

[8] Bi X , Sun J, Ji AX, Baudry M. Potential therapeutic approaches for Angelman syndrome. Expert Opin Ther Targets. 2016;20:601–613. 26558806 10.1517/14728222.2016.1115837 PMC4902328

[9] Meng L , Ward AJ, Chun S, Bennett CF, Beaudet AL, Rigo F. Towards a therapy for Angelman syndrome by targeting a long non-coding RNA. Nature. 2015;518:409–412. 25470045 10.1038/nature13975 PMC4351819

[10] Milazzo C , Hoener MC, Elgersma Y, et al Antisense oligonucleotide treatment rescues UBE3A expression and multiple phenotypes of an Angelman syndrome mouse model. JCI Insight. 2021;6:e145991. 10.1172/jci.insight.145991 PMC8410092 34369389

[11] Sidorov MS , Deck GM, Dolatshahi M, et al Delta rhythmicity is a reliable EEG biomarker in Angelman syndrome: A parallel mouse and human analysis. J Neurodev Disord. 2017;9:1–14. 28503211 10.1186/s11689-017-9195-8 PMC5422949

[12] Frohlich J , Miller MT, Bird LM, et al Electrophysiological phenotype in angelman syndrome differs between genotypes. Biol Psychiatry. 2019;85:752–759. 30826071 10.1016/j.biopsych.2019.01.008 PMC6482952

[13] Martinez LA , Born HA, Harris S, et al Quantitative EEG analysis in angelman syndrome: Candidate method for assessing therapeutics. Clin EEG Neurosci. 2020:155005942097309. 10.1177/1550059420973095 33203220

[14] Ostrowski LM , Spencer ER, Bird LM, et al Delta power robustly predicts cognitive function in Angelman syndrome. Ann Clin Transl Neurol. 2021:8;1433–1445. 34047077 10.1002/acn3.51385 PMC8283185

[15] Hipp JF , Frohlich J, Keute M, Tan W-H, Bird LM. Electrophysiological abnormalities in angelman syndrome correlate with symptom severity. Biol Psychiatry Glob Open Sci. 2021;1:201–209. 34841387 10.1016/j.bpsgos.2021.05.003 PMC8622755

[16] Klem GH , Lüders HO, Jasper HH, Elger C. The ten-twenty electrode system of the International Federation. The international federation of clinical neurophysiology. Electroencephalogr Clin Neurophysiol. 1999;52:3–6. 10590970

[17] Bokil H , Andrews P, Kulkarni JE, Mehta S, Mitra PP. Chronux: A platform for analyzing neural signals. J Neurosci Methods. 2010;192:146–151. 20637804 10.1016/j.jneumeth.2010.06.020 PMC2934871

[18] Swayze EE , Siwkowski AM, Wancewicz E V, et al Antisense oligonucleotides containing locked nucleic acid improve potency but cause significant hepatotoxicity in animals. Nucleic Acids Res. 2007;35:687–700. 17182632 10.1093/nar/gkl1071 PMC1802611

[19] Chu CJ , Leahy J, Pathmanathan J, Kramer MA, Cash SS. The maturation of cortical sleep rhythms and networks over early development. Clin Neurophysiol. 2014;125:1360–1370. 24418219 10.1016/j.clinph.2013.11.028 PMC4035415

[20] Yu Z , Guindani M, Grieco SF, Chen L, Holmes TC, Xu X. Beyond t test and ANOVA: Applications of mixed-effects models for more rigorous statistical analysis in neuroscience research. Neuron. 2022;110:21–35. 34784504 10.1016/j.neuron.2021.10.030 PMC8763600

[21] Germaine ND , Gorka D, Drennan R, et al Antisense oligonucleotides targeting UBE3A-ATS restore expression of UBE3A by relieving transcriptional interference. bioRxiv 2021.

