# Supplementary figures and images for: Longitudinal EEG model detects antisense oligonucleotide treatment effect and increased UBE3A in Angelman syndrome
Source: Brain Commun. 2022 Apr 26;4(3):fcac106. doi: 10.1093/braincomms/fcac106 (PMC9123847; doi:10.1093/braincomms/fcac106)

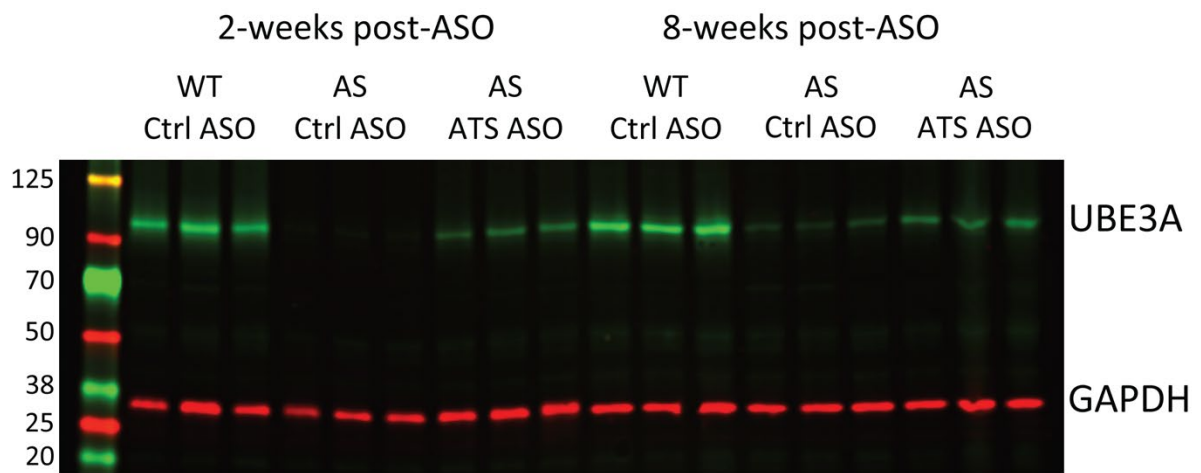

Supplement: fcac106_Supplementary_Data [file fcac106_supplementary_data.pdf]
